# Building capacity for mental health resilience – local impact of the UK Better Mental Health Fund

**DOI:** 10.1093/eurpub/ckac131.480

**Published:** 2022-10-25

**Authors:** M Lie, S Visram

**Affiliations:** Population Health Sciences Institute, Newcastle University, Newcastle upon Tyne, UK

## Abstract

**Background:**

COVID-19 as a public mental health emergency has exacerbated existing mental health inequalities. The UK government invited local authorities with areas of high deprivation to apply for a year of funding, in order to address the mental health impacts of COVID-19 and incentivize investment in prevention and promotion interventions for better mental health. South Tyneside Council in North East England made a successful bid to the Better Mental Health Fund (BMHF), and distributed grants to 7 organisations delivering 13 programs. A qualitative evaluation of these programs aimed to answer the following questions:

1. How were the funded programs implemented?

2. What difference did they make to local beneficiaries?

3. How might these programs and their impacts be sustained into the future?

4. Has the BMHF led to any wider impacts on organisations and local communities?

**Methods:**

In-depth interviews with individuals, pairs and groups were conducted online or in person with service providers and beneficiaries. Non-verbatim transcripts were made from recordings, checked with verbatim transcripts from Teams and Zoom, and analyzed thematically to generate a coding frame. Throughout the analysis, comparisons were made between organizations and programs.

**Results:**

Fifteen interviews involving 22 participants lasting up to an hour each were conducted. The main themes identified as impactful were 1) community approaches based on supportive and good relationships between the local authority public health lead and participating organizations (mainly voluntary agencies), enabling 2) capacity-building for mental health resilience and 3) community empowerment. This was despite the short turnaround of the grant application process, limited time to deliver on targets, and anxieties about future sustainability.

**Conclusions:**

Short-term funding can build capacity in mental health resilience in deprived areas if administered by public health leaders who relate well with provider organizations.

**Key messages:**

• Public health leaders who relate well with provider organizations are key drivers of community health promotion strategies that include mental health capacity building.

• Qualitative methods used in evaluations can inform public health commissioning by capturing the benefits and challenges of short-term funding for interventions promoting community mental health.

